# Personality and performance are affected by age and early life parameters in a small primate

**DOI:** 10.1002/ece3.3833

**Published:** 2018-04-15

**Authors:** Pauline B. Zablocki‐Thomas, Anthony Herrel, Isabelle Hardy, Lucile Rabardel, Martine Perret, Fabienne Aujard, Emmanuelle Pouydebat

**Affiliations:** ^1^ Département Adaptations du Vivant UMR 7179 C.N.R.S/M.N.H.N. Paris Cedex 5 France; ^2^ Evolutionary Morphology of Vertebrates Ghent University Ghent Belgium

**Keywords:** early life, life history, performance, personality, phenotypic correlation

## Abstract

A whole suite of parameters is likely to influence the behavior and performance of individuals as adults, including correlations between phenotypic traits or an individual's developmental context. Here, we ask the question whether behavior and physical performance traits are correlated and how early life parameters such as birth weight, litter size, and growth can influence these traits as measured during adulthood. We studied 486 captive gray mouse lemurs (*Microcebus murinus*) and measured two behavioral traits and two performance traits potentially involved in two functions: exploration behavior with pull strength and agitation score with bite force. We checked for the existence of behavioral consistency in behaviors and explored correlations between behavior, performance, morphology. We analyzed the effect of birth weight, growth, and litter size, while controlling for age, sex, and body weight. Behavior and performance were not correlated with one another, but were both influenced by age. Growth rate had a positive effect on adult morphology, and birth weight significantly affected emergence latency and bite force. Grip strength was not directly affected by early life traits, but bite performance and exploration behavior were impacted by birth weight. This study shows how early life parameters impact personality and performance.

## 1 INTRODUCTION

Behavior and physical performance are considered integrative traits as they are impacted by a number of other traits and are acknowledged to have an important role on an individual's fitness, especially in continuously changing and challenging environments (van Valen, 1973). Performance is the ability of an organism to execute an ecologically relevant task (Huey & Stevenson, 1979) and is the organismal trait that provides a direct link between morphology and fitness (Arnold, 1983). Physical performance traits can have direct benefits for animals, for example in the case of Darwin's finches where bite force is directly related to resource exploitation (Herrel, Podos, Huber, & Hendry, 2005).

Behavioral traits also impact fitness, but are often considered to be more labile phenotypic traits at the level of the individual (Kappeler & Kraus, 2010). Indeed, individuals are capable of adapting their behavior according to the situation they have to cope with (Dingemanse, Kazem, Réale, & Wright, 2010; Piersma & Drent, 2003). However, this intra‐individual plasticity in behavior also presents limitations, when individuals do not adapt their behavior to the context (Pruitt, Riechert, & Jones, 2008), which may have negative fitness consequences. For example, some female spiders (*Anelosimus studiosus*) are very aggressive not only during foraging but also in mating contexts. These limitations are commonly referred to as “behavioral syndromes” (Sih, Bell, & Johnson, 2004) when individuals present a fixed “behavioral type” in every situation. This is also referred to as “animal personality” (Réale, Reader, Sol, McDougall, & Dingemanse, 2007) when individuals tend to present a personal and consistent reaction in a specific context and throughout their lives (Stanley, Mettke‐hofmann, & Preziosi, 2017). More specifically, personality is generally defined as “consistent individual behavioral differences” (Réale & Dingemanse, 2012; Réale et al., 2007), and behavioral syndromes are “a suite of correlated behaviors expressed within a context or across different contexts” (Sih, Bell, Chadwick Johnson, & Ziemba, 2004; Sih, Bell, et al., 2004). In animals, personality has been described along a set of five axes including shyness/boldness, aggressiveness, activity, sociality, and exploration (Réale et al., 2007). These five factors are derived from the big five factors in human personality (Gosling 2001). Nowadays, the field of animal personality is very important in the study on animal behavior, and researchers are investigating its evolutionary relevance (Beekman & Jordan, 2017; Briffa, 2017). Yet, there is also a need to explore the proximate determinants and the development of individual behavioral differences (Stamps & Groothuis, 2010; Stanley et al., 2017).

However, limitations on animal personality can appear, rending the response of the individuals more predictable. For example, age is a factor that tends to limit flexibility of individuals in some mammals, as gray mouse lemurs (*Microcebus murinus*) and marmots (*Marmota flavivenris*) (Dammhahn, 2012; Petelle, McCoy, Alejandro, Martin, & Blumstein, 2013), and sex can account for differences in personality in association with ecological difference between sexes (Dammhahn, 2012). Finally, personality can be linked to other phenotypic traits such as metabolism or performance, as they limit the range of possible behaviors available to the individual (Careau & Garland, 2012).

A simple way to understand limitations in integrative phenotypic traits like personality and physical performance is to detect whether they share a relationship with other traits. As explained by Falconer and Mackay (1996), the study of the correlations between parameters is important for three main reasons: (1) detecting the effect of pleiotropic genes (i.e., genes that impact several phenotypic traits), (2) detecting correlated selection between traits, and (3) detecting the relationship between the traits and associated fitness. A first and direct way to study the correlation between traits is to conduct phenotypic correlations. This is commonly understood as the ratio between trait covariance and the product of the standard deviations of the phenotypic values. Phenotypic correlations are an expression of the combination of genetic and environmental correlations between traits (Falconer & Mackay, 1996). Indeed, as behavior and performance are integrative traits, they can show correlational selection with other traits potentially involved in a common function and have overall fitness consequences (Careau & Garland, 2012; Réale et al., 2007).

However, variation in behavior, morphology, and performance can also be the result of determinants early in life linked to the environment (Petelle et al., 2013; Riley et al., 2017; Schuett, Dall, Wilson, & Royle, 2013), or directly linked to the individual and its early life context (Rödel, Bautista, García‐Torres, Martínez‐Gómez, & Hudson, 2008; Rödel, Bautista, Roder, Gilbert, & Hudson, 2017). In rabbits, for example, individuals that were born heavier also had higher growth rates than others by having a higher food intake and a more efficient conversion of food into energy for growth. This influenced their development later in life, possibly impacting fitness (Rödel et al., 2008). In the same species the difference in body mass gain between siblings was also correlated with differences in behavior, with lighter individuals being less docile and having a lower exploration activity than heavier littermates (Rödel et al., 2017). In mouse lemurs, individuals born with lower body mass start exploration later in an open‐field test than do individuals with a higher body mass at birth (Thomas, Herrel, Hardy, Aujard, & Pouydebat, 2016). The two commonly adopted explanations for the impact of early life parameters on behavioral differences are first, that the conditions in early life provide insights into the environment for later in life, and second, that the conditions experienced at early stages have effects on an individual's somatic state that last throughout its life (Nettle & Bateson, 2015). Food restriction, for example, could induce behavioral types that are more prone to searching for food, individuals that are more aggressive, and individuals with smaller body sizes (Dirienzo & Montiglio, 2016).

The aim of the present study was to combine the investigation of behavioral and physical performance limitations through the study of phenotypic correlations between traits in adults. We specifically tested the impact of early and current life parameters on adult phenotypes. To do so, we took advantage of a large colony of captive gray mouse lemurs, in which animals are monitored from birth to death. This small primate is an arboreal and nocturnal species that reproduces once a year, that is amenable for measurements of morphology and performance, and in which personality has already been described in captivity (Thomas, Herrel, et al., 2016) and in the wild (Dammhahn & Almeling, 2012). We studied two performance traits and two behaviors that could be functionally related: bite force and agitation score on the one hand, and pull strength and emergence latency on the other hand. We deliberately studied simple measurements of behavior in order to be able to measure a large number of individuals (*N* = 486). We investigated the effect of three early life parameters: body mass at birth, growth rate, and litter size on the previously described behavioral and performance traits.

## 2 METHODS

### 2.1 Subjects

We collected data for 486 different gray mouse lemurs (*Microcebus murinus*) in a captive colony of mouse lemurs. Individuals are housed in large cages in groups of three or four individuals. Ambient air temperature is maintained at 25°C and humidity is stable at around 30%. All individuals are fed ad libitum, weighed monthly, and maintained under artificial light conditions mimicking natural seasons.

### 2.2 Phenotypic traits

#### 2.2.1 Physical performance

##### Pull strength

We used a small iron bar mounted onto a piezoelectric force platform (Kistler squirrel force plate, ±0.1°N; Winterthur, Switzerland) connected to a charge amplifier (Kistler charge amplifier type 9865) as described in Thomas et al. (2015). We conducted tests for 60 s and recorded forces at 1 kHz. We let the animal grab the bar and pulled it away from the bar horizontally. A singe recording session included several pulls and three recording sessions were performed for each individual. We then extracted the maximum force across all sessions using the BioWare software (Kistler).

##### Bite force

We used a piezoelectric transducer (Kistler, type 9203, range ± 500°N; Kistler, Winterthur, Switzerland) attached to a handheld charge amplifier (Kistler, type 5995) to record bite force. The transducer was placed between two plates that animals had to bite, as described in (Herrel, Spithoven, Van Damme, & De Vree, 1999) (see also Chazeau, Marchal, Hackert, Perret, & Herrel, 2013; Thomas et al., 2015 for studies on mouse lemurs). We covered plates with a layer of cloth medical tape to provide grip on the plates and to protect their teeth. Next, we adapted the distance between plates to the size of the lemurs so that we could measure bite force during unilateral molar biting where bite force is maximum in mammals (Dumont & Herrel, 2003). We conducted three recording sessions, and only the highest bite force across the three sessions was kept for the analysis.

#### 2.2.2 Behavioral recordings

##### Emergence tests

We conducted emergence tests using a small wooden box (18 × 18 × 31 cm). We caught animals directly in their nest box between 1:00 to 5:00 pm, identified animals, and placed a single individual in the wooden box. Next, we placed the wooden box at the entrance of the home cage of the individual. We then waited at least 2 min so that the animal could habituate and calm down from the manipulation. The test consisted of opening the trap door and recording the latency for the animal to escape from the box and return to its home cage. The test lasted 5 min maximum. Individuals that never left the box within the allotted 5 min were given a score of 300 s. We conducted this test between 1 and 13 times per individual for a total of 1,238 tests. Some individuals were tested only once as they died before we could test them twice (note that these individuals were included in our analysis). Fifty‐eight individuals were tested over 2 years in summer in order to explore the long‐term repeatability. We waited at least 3 weeks before repeating the test with the same individual.

##### Agitation score

We followed the same protocol as described in (Verdolin & Harper, 2013) testing each animal between one and six times for a total of 1,001 tests. In brief, the test consisted in grabbing the animal and scoring its reaction: urinating (1 point), defecating (1 point), screaming (1 point), struggling (2 points), and biting (3 points). According to this protocol, animals were given a score from 0 to 8. The scoring started directly after extraction of the animal from its nest box and lasted 30 s maximum. We rated agitation occasionally during different events of the monitoring protocols: when animal keepers conducted the monthly weighing or before physical testing.

#### 2.2.3 Morphology

We recorded the length of metatarsus, tibia, ulna, head width, head length, and head depth with a digital calliper (±0.01 mm; Mitutoyo, Kanagawa, Japan). Body weight at the time of each test was extracted from the colony database.

#### 2.2.4 Life history traits

##### Early life data

We extracted body weight at birth, body weight at 3 months, and body weight at testing, litter size, and mother identity from the colony data base. Growth rate was calculated as the weight gain in grams over during the first 3 months of life, which is the period during which most of the growth occurs in this species (Castanet et al., 2004).

### 2.3 Statistics

Physical performance and behavioral variables were log_10_‐transformed to render the data normal and homoscedastic. All the statistics were performed in R version 3.3.3 (2017‐03‐06). We considered that an effect was statistically significant when its *P*‐value was below the threshold of α = 0.05.

#### 2.3.1 PCA on morphological traits

We considered the first component of a PCA performed on the morphological traits as a marker of global body size. This axis explained 40% of the variance of the six morphological dimensions and was positively correlated with all dimensions (head and limbs). PCA scores were not log_10_‐transformed.

#### 2.3.2 Repeatability

We used rptR package to estimate repeatability of behavioral traits (Nakagawa & Schielzeth, 2010) as a verification of the consistency in behavior (i.e., the intraclass correlation coefficient). We tested between‐year repeatability and within‐year repeatability for emergence latency as some individuals were tested over longer periods of time (Biro & Stamps, 2015).

#### 2.3.3 Effect of early life parameters on adult phenotype

We ran linear mixed modeling with lme4 package (Bates et al., 2014).

We conducted regressions of birth parameters taken separately on phenotypic traits of adults. As a response factor, we separately considered pull strength, bite force, emergence latency, agitation score, and morphology. As fixed effects, we added: body mass at birth, growth rate during the first 3 months of life, and litter size. We systematically added age, sex, and body weight as covariates because of the presence of animals of different ages, the presence of a sexual dimorphism in this species (Kappeler, 1991), and an effect of body weight on physical performance (Thomas, Pouydebat, Brazidec, Aujard, & Herrel, 2016; Thomas et al., 2015). In addition, as a quadratic effect of age is expected for physical performance (Berthelot et al., 2012), we added age squared as a covariate in models for grip strength and bite force. For morphology, we did not consider body weight as a fixed effect as it is not expected to impact morphology. As a random effect, we added individual identity for response variables presenting repeated measurements (behavior and performance), mother identity, and the animal room where they were raised to account for permanent environmental effects.

### 2.4 Ethical note

All subjects included in the study were born and reared in captivity. All experiments were approved by and in accordance with the guidelines of the local institutional ethics committee.

## 3 RESULTS

### 3.1 Repeatability of behavioral measures

#### 3.1.1 Emergence latency

Emergence latency was repeatable on short timescales (a 3‐week interval, *R* = 0.33 ± 0.04 *SE* (CI = [0.254, 0.405], *p* < .01). For animals that were tested 1 year apart, average emergence latency was also repeatable between years (*R* = 0.29 ± 0.11 *SE* (CI = [0.057, 0.51], *p* = .014).

#### 3.1.2 Agitation score

The agitation score was repeatable (*R* = 0.28 ± 0.04 (CI = [0.207, 0.357], *p* = 0.01). When we added mother identity as a fixed effect, repeatability dropped (*R* = 0.16 ± 0.05 *SE* (CI = [0.169, 0.382], *p* = .23) and lost significance, suggesting an important maternal effect on this behavior. Mother effect was then taken in consideration in following statistics as a random effect.

### 3.2 Effect of early and current life parameters on personality and performance

Both personality traits were not correlated with one another and were not correlated with the performance traits measured. (Table S1). Litter size was negatively correlated with birth weight (*r* = −.36, *p* < .001), and growth rate (*r* = .13, *p* = .04) was not correlated with birth weight (Table S2).

When we tested for the effect of three early life parameters on physical performance, behavior, and morphology, we found an effect of birth weight on morphology (β = 1.29 ± 0.3, *p* < .001), bite force (β = 0.10 ± 0.05, *p* = .04), and emergence latency (β = 0.67 ± 0.3, *p* = .04), with individuals becoming larger and having higher bite force and a longer emergence latency in adulthood if they were heavier at birth (Table 1).

**Table 1:** Summary of the effect of birth parameters on phenotypic traits

	Estimate	*SE*	*t*‐Value	*p*‐Value
Grip strength				
Birth weight (g)	0.055	0.041	1.324	.186
Growth rate (g/day)	0.088	0.049	1.800	.072
Litter size	0.000	0.013	0.018	.986
Age (days)	**0**.**733**	**0**.**191**	**3**.**828**	**<**.**001**
Age^2^	−**0**.**056**	**0**.**014**	−**3**.**919**	**<**.**001**
Sex—Males	−0.009	0.018	−0.488	.626
Body weight (g)	**0**.**287**	**0**.**050**	**5**.**726**	**<**.**001**
Bite force				
Birth weight (g)	**0**.**105**	**0**.**052**	**2**.**013**	.**044**
Growth rate (g/day)	−0.032	0.061	−0.531	.595
Litter size	0.017	0.016	1.049	.294
Age (days)	**0**.**654**	**0**.**243**	**2**.**691**	.**007**
Age^2^	−**0**.**047**	**0**.**018**	−**2**.**628**	.**009**
Sex—Males	−**0**.**044**	**0**.**020**	−**2**.**149**	.**032**
Body weight (g)	**0**.**445**	**0**.**051**	**8**.**657**	**<**.**001**
Emergence latency				
Birth weight (g)	**0**.**669**	**0**.**321**	**2**.**081**	.**037**
Growth rate (g/day)	−0.709	0.379	−1.872	.061
Litter size	−0.092	0.099	−0.928	.354
Age (days)	−0.383	0.083	−4.600	**<**.**001**
Sex—Males	−0.148	0.137	−1.086	.277
Body weight (g)	0.440	0.311	1.413	.158
Agitation score				
Birth weight (g)	0.123	0.164	0.749	.454
Growth rate (g/day)	−0.111	0.186	−0.600	.549
Litter size	0.024	0.049	0.495	.620
Age (days)	−0.218	0.042	−5.173	**<**.**001**
Sex–Males	−0.174	0.066	−2.618	.009
Body weight (g)	−0.095	0.154	−0.619	.536
Morphology				
Birth weight (g)	**1**.**293**	**0**.**336**	**3**.**842**	**<**.**001**
Growth rate (g/day)	**3**.**319**	**0**.**377**	**8**.**793**	**<**.**001**
Litter size	−0.015	0.104	−0.142	.887
Age (days)	**0**.**860**	**0**.**088**	**9**.**826**	**<**.**001**
Sex–Males	−0.158	0.128	−1.236	.216

Bolded values represent statistically significant effects.

**Figure 1:**
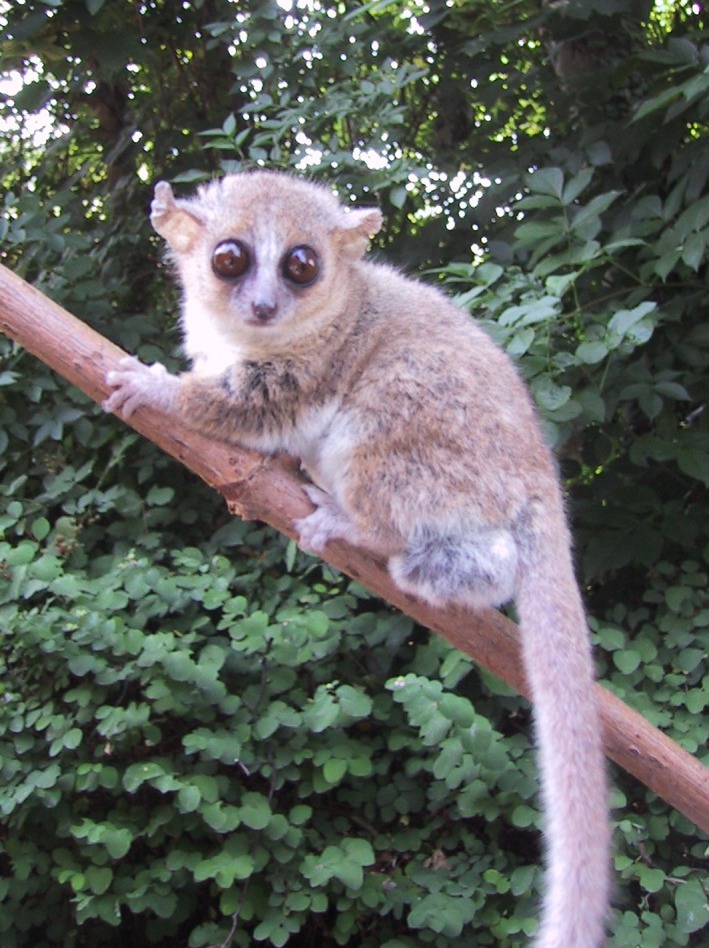
Photograph of a male gray mouse lemur in the captive colony of Brunoy

Growth rate had a positive impact on morphology (β = 3.32 ± 0.4, *p* < .001), with higher growth rates leading to larger individuals. Growth rate also tended to influence pull strength positively (β = 0.088 ± 0.05, *p* = .07) and emergence latency negatively (β = −0.71 ± 0.06, *p* = .06), with individuals that grew less having longer emergence latencies than individuals that grew more. We found no detectable effect of litter size in these models (Figure 1).

Our models showed that age had a marked linear impact on both behavioral traits: younger individuals had shorter emergence latency (β = −0.38 ± 0.08, *p* < .001) and higher agitation scores (β = −0.22 ± 0.04, *p* < .001) than older individuals. Age also had a significant negative quadratic effect on both grip strength (β = −0.06 ± 0.01, *p* < .001) and bite force (β = −0.047 ± 0.02, *p* < .01), and a linear positive effect on morphology (β = 0.86 ± 0.09, *p* < .001), with animals becoming larger with age (Table 1).

Sex influenced bite force and the agitation score. Male ones were less agitated (β = −0.17 ± 0.06, *p* < .01) and bit less hard (β = −0.04 ± 0.02, *p* = .03) than female ones. However, we did not find differences in overall size between the sexes. Body weight also had a positive impact on both grip strength (β = 0.29 ± 0.05, *p* < .001) and bite force (β = 0.45 ± 0.05, *p* < .001).

## 4 DISCUSSION

In this study, we showed that behavioral and physiological traits are influenced by early life parameters in different ways. Indeed, we found that a behavior such as emergence latency was influenced directly by early life parameters and especially by body weight at birth, as was bite force performance. However, pull strength was more determined by morphology, which, in turn, was influenced by early life parameters including birth weight and growth rate.

We found that behavioral variables were repeatable between years and within years for emergence latency, accounting for a low temporal plasticity. Agitation score was also repeatable, but largely determined by maternal effects. For this trait, maternal identity seemed to be one of the principal limitations in personality. We did not test for temporal plasticity (Biro & Stamps, 2015), however. The repeatability of the behavioral traits measured here was similar to that of personality traits measured in other studies, including ones on birds (0.27‐0.66) or mouse lemurs (0.16–0.45) (Dammhahn & Almeling, 2012; Dingemanse, 2002).

We found no relation between the two behavioral variables and could not conclude in favor of the presence of a behavioral syndrome. This also suggests that these two personality traits did not interact with one another. Agitation scores in mouse lemurs have previously been correlated with their response to human handling (Verdolin & Harper, 2013). In that study, the authors suggested that the agitation score may be interpreted as shyness or anxiety. In our study, mean agitation was also positively correlated with heart rate (Table S1), which could thus suggest that the agitation score was a good indicator of stress during manipulation in this species. Thus, the absence of a correlation between emergence latency and the agitation score confirmed that these two consistent behaviors illustrate two different personality axes, presumably exploration and boldness/shyness.

In this study, we found an effect of birth weight on morphology, bite force, and emergence latency, which is consistent with the expectation of Nettle and Bateson (2015) who argued that early life inputs could alter an individual's phenotype. Individuals born with a lower birth weight had a shorter emergence latency. This observation is consistent with a result previously found for this species in open‐field tests, where individuals born with a lower body weight started exploring earlier (Thomas, Herrel, et al., 2016). It was further proposed that small newborns could benefit more from exploring the environment sooner to avoid competition. This interpretation seems to be confirmed by the fact that individuals with a shorter emergence latency tend to have higher growth rates. However, we did not detect correlations between birth weight and growth rate, meaning that individuals that were born with a lower birth weight were not the ones that will grow most. Birth weight affected bite force but not grip strength. Early life parameters also had a strong effect on body size. However, we found no correlation between birth weight and growth rate (Supporting information), which is different of what is observed in other species, such as rabbits (Rödel et al., 2008), indicating that birth weight is not a principal determinant for growth. Finally, birth weight was negatively correlated with litter size, as is commonly observed in mammals (Tuomi, 1980) including captive gray mouse lemurs (Perret, 1982).

We also considered possible sources of limitations in the variability in behavior and performance. Body mass, for example, had a large influence on physical performance when accounting for age and sex. This could be explained by the important correlation between body size and body weight (Thomas, Pouydebat, Brazidec, et al., 2016; Thomas, et al., 2015), but also by a higher body condition (with heavier individuals having more fat resources – more access to food). In mouse lemurs, morphology was also affected by age, as individuals continue to grow at least until 6–7 years (Castanet et al., 2004). In our dataset, individuals rarely surpassed 7 years, as the mean life expectancy of this species in captivity is on average 5–6 years, independent of sex (Perret, 1997).

We found that age influenced emergence latency and agitation score, showing that adult personality is subject to change during an individual's life span, sometimes described as “temporal plasticity” (Stamps & Biro, 2016). This is consistent with a study in wild mouse lemurs, showing that personality changed with age class, with older individuals being bolder and taking more risk that younger ones (Dammhahn, 2012), at least in males. In that study, they proposed that young males are less prone to take risks, as they have not yet reproduced. This could be also the case in the captive condition of the laboratory where competition for females is also high, and where only one dominant male (over groups of three males) usually sires all the offspring (Andrès, Solignac, & Perret, 2003). We only presented models testing for a linear effect of age on personality, as we could more probably expect a linear change in personality with age in this species (Dammhahn, 2012). Models including a quadratic effect of age on personality showed no significant effect of age on personality, and Akaike information criteria were lower for models with only a linear effect of age (results not shown). However, as expected, there was a quadratic effect of age on physical performance, describing an “inverted U shape” of performance in relation to age (Berthelot et al., 2012).

Sex differences may also be a source of limitation in behavior (Kappeler & Kraus, 2010) and physical performance (Law, Venkatram, & Mehta, 2016). Here, we found an effect of sex on bite force and agitation scores. In this dataset, females bit harder than males, when age and body mass were taken into account (Chazeau et al., 2013). This result is presumably explained by the known head size dimorphism (Thomas et al., 2015). These models did not extract sexual dimorphism in morphology as we could have expected it, but simple regression of sex on morphology showed that males are smaller than females when controlling for age (β_male_ = −0.35 ± 0.14 *SE*,* p* = .01)*,* suggesting that the effect or early life parameters had a higher impact on morphology than sexual dimorphism. Indeed, even if males were slightly heavier at birth (males = 6.6 g ± 1.2 *SD*; females = 6.5 g ± 1.3 *SD*), females grew more during their first 3 month of life (males = 61.5 g ± 8.1 *SD*; females = 65.8 g ± 9.9 *SD*) and had a higher growth rate (when accounting for mother and animal housing as random effects; β_male_ = −0.065 ± 0.015 *SE*,* p* < .001).

In conclusion, limitations in phenotypic variability can appear for various reasons in a population. Here we showed that early life parameters had both direct and indirect effects on the adult phenotype in a captive population of gray mouse lemurs. Birth weight had an impact on adult behavior and on bite force, which seems convergent with the hypothesis that early life impacts somatic state. Growth rate influenced adult performance through an effect on adult morphology. Taken together, these data show that limitations of behavioral variability and physical performances are the result of both early and current factors.

## CONFLICT OF INTEREST

None declared.

## AUTHORS CONTRIBUTIONS

Data were collected by the first author with the help of the fourth author, with the support of animal keepers from the colony. Long‐term data from the colony database were collected by animal keepers and the fifth author since the establishment of the colony and were recently assembled and monitored by the third author. Data analysis and writing were conducted by the first author with the help of the second, sixth, and last author.
