# Supplementary material for: Personality and performance are affected by age and early life parameters in a small primate
Source: Ecol Evol. 2018 Apr 15;8(9):4598–605. doi: 10.1002/ece3.3833 (PMC5938443; doi:10.1002/ece3.3833)
Supplement: Supplementary file 1 [file ECE3-8-4598-s001.docx]

# **Supporting Information**

## Phenotypic correlations between behavioural and performance traits

A simple way to understand limitations in integrative phenotypic traits such as personality and physical performance is to detect if they share a relationship with other traits. As explained by Falconer and Mackay (1996), the study of the correlations between parameters is important for three main reasons: (1) detecting the effect of pleiotropic genes (i.e. genes that impact several phenotypic traits.), (2) detecting correlated selection between traits, and (3) detecting the relationship between the trait and associated fitness. A first and direct way to study the correlation between traits is conducting phenotypic correlations. This is commonly understood as the ratio between trait covariance to the product of the standard deviations of the phenotypic values. This phenotypic correlation is an expression of the combination of genetic and environmental correlations between traits. Correlations between personality and other phenotypic have been described in several species, and include morphology in capuchin monkeys (*Cebus paella*) (Wilson et al. 2014) or spiders (*Stegodyphus dumicola*) (Wright, Keiser, and Pruitt 2015), or performance in zebrafish (*Danio rerio*) (Kern et al. 2016). In grey mouse lemurs (*Microcebus murinus*) smaller individuals had shorter starting latency in the exploration of an open field test environment than larger individuals (Thomas et al. 2016). Indeed, as behaviour, but also performance both are integrative traits, they can show correlational selection with other traits potentially involved in a common function and have overall fitness consequences (Réale et al. 2007; Careau and Garland 2012). Relationships between traits can also appear through the effect of pleiotropic genes (Kern et al. 2016), and can link performance with behaviour and other performance traits. This can then lead to phenotypic correlations between traits that are involved in different functions. Phenotypic correlations are thus interesting from an evolutionary perspective as they presents a similar pattern of variation as do genetic correlations, balanced by the effects of the environment (Cheverud 1988).

### Methods

#### Correlation between phenotypic traits

We tested the correlation between behavioural, performance, and morphological traits. We averaged the behavioural variables for each individual and compared them in a correlation matrix with Pearson’s correlations (rcorr function from Hmisc package). Second, we tested the correlation between the three early life parameters (birth weight, growth rate, and litter size). For females, we tested for correlations between reproductive success, behavioural traits, performance, and morphological traits. We conducted Bonferroni corrections for multiple testing.

### Heart rates

We used a microphone (Tascam DR-05) to record heart rates in mp3 format by placing the microphone on the chest of the animal during one minute. For counting heart rates, we used Audacity software and counted the number of heart beats and multiplied them by two to obtain the number of heart beats per minute.

### Results

We found that our agitation score was positively correlated with heart rate, with less agitated individuals having lower heart rates than more agitated individuals. Morphological and physical performance traits were also positively correlated (**Supplementary Table 1**). When we applied Bonferroni correction for these 15 correlations, α = 0.003 had to be considered as the threshold for significance. Under this condition, these correlations were still significant.

**Supplementary Table 1**: Correlation matrix summarizing correlations between behavioural, performance, and morphological variables.

|  | Emergence Latency (s) | Heart rate (cycle/min) | Bite force (N) | Pull strength (N) | Morphology (PCA1) |
| --- | --- | --- | --- | --- | --- |
| *N* | 284 | 283 | 284 | 284 | 277 |
| Agitation score | 0.04  P=0.46 | **0.18**  **P=0.0024** | -0.01  P=0.54 | -0.01  P=0.87 | -0.09  P=0.12 |
| Emergence Latency (s) |  | -0.03  P=0.67 | -0.10  P=0.10 | -0.09  P=0.12 | -0.09  P=0.12 |
| Heart rate (cycle/min) |  |  | 0.01  P=0.85 | 0.10  P=0.10 | -0.04  P=0.52 |
| Bite force (N) |  |  |  | **0.30**  **P<0.001** | **0.30**  **P<0.001** |
| Pull strength (N) |  |  |  |  | **0.30**  **P<0.001** |

Bolded values represent significant correlations.

Litter size was negatively correlated with birth weight, and positively correlated with growth rate, with larger litter sizes being composed of lighter individuals that grew more during the first three months of their life. However, when we applied a Bonferroni correction for these 3 correlations (α = 0.017) the relationship between litter size and growth rate lost significance. We found no significant correlation between birth weight and growth rate (**Supplementary Table 2**).

**Supplementary Table 2**: Correlation matrix summarizing correlations between early life parameters.

|  | Birth weight | Growth rate | Litter size |
| --- | --- | --- | --- |
| N | 258 | 235 | 258 |
| Birth weight |  | -0.06  P = 0.34 | **-0.36**  **P < 0.001** |
| Growth rate |  |  | **0.13**  **P = 0.04** |

Bolded values represent significant correlations.

None of the early life parameters, nor sex, age or body weight were correlated with heart rate (**Supplementary Table 3**).

**Supplementary Table 3**: Summary of the effect of early life parameters on heart rate.

|  | Estimate | S.E. | *t* | *P* |
| --- | --- | --- | --- | --- |
| Heart Rate *N=328*  $\bar{\boldsymbol{X}}$ *± SD: 257.1 ± 19 cycle/min* | | | | |
| Birth weight (g) | 0.0166 | 0.0235 | 0.71 | 0.48 |
| Growth rate (g/day) | 0.0245 | 0.02841 | 0.86 | 0.39 |
| Litter size | 0.00655 | 0.00704 | 0.93 | 0.35 |
| Age (days) | -0.00842 | 0.00618 | -1.36 | 0.17 |
| Sex - Males | -0.0154 | 0.00964 | -1.61 | 0.11 |
| Body weight (g) | -0.0239 | 0.0265 | -0.90 | 0.37 |
